# Cod, construction, and communities: Relations between fish and architectural history in Ísafjörður, Iceland

**DOI:** 10.12688/openreseurope.20177.3

**Published:** 2025-09-22

**Authors:** André Tavares, Karl Benediktsson, Ana Azevedo, Rafael Sousa Santos, Garðar Eyjólfsson, Michelle Valliant

**Affiliations:** 1 Center for Studies in Architecture and Urbanism, Faculty of Architecture of the University of Porto, Porto, Porto District, Portugal; 2 Faculty of Life and Environmental Sciences, University of Iceland, Reykjavík, Capital Region, Iceland; 3 Research Centre West Fjords, University of Iceland, Research Centre of the Westfjords, Bolungarvík, Iceland

**Keywords:** cod life cycle; environmental studies; fisheries; marine ecology; research methods; urban development; urban history

## Abstract

Understanding the interplay between ecological processes and human-built environments is key to addressing the entangled dynamics of marine and terrestrial landscapes, yet these relationships are often studied separately. This paper addresses this issue by exploring the socioecological histories of North Atlantic fishing landscapes, with a focus on Ísafjörður, situated in the Westfjords of Iceland, a critical site in the history of cod ( *Gadus morhua*) fisheries.

It presents the outcomes of a weeklong workshop that explored the connections between urban development and cod populations through three thematic lenses: 1) the urban development of Ísafjörður, 2) the life cycle of cod, and 3) Icelandic fisheries. A range of interdisciplinary methods was applied, including archival photography analysis, historical mapping, cod movement pattern reconstruction, oceanographic data assessment, investigation of vessel technology, and fisheries data analysis.

Our findings reveal overlapping timelines that link fish population cycles with patterns of urban growth, highlighting points of convergence where ecological and social dynamics intersect. Visual representation was used as an integrative tool to translate between marine processes and the built environment. By reflecting on the workshop’s experiments and achievements, this paper proposes strategies for future research at the intersection of ecology, fisheries, and architecture.

## Introduction

Research has acknowledged the relationship between fishing pressure and the health of fish populations, including demersal fish such as cod ( Thurstan *et al.*, 2010; Thurstan, 2022). While there are several factors that determine the size of catches—including environmental conditions, the fishing gear used, and the degree of effort involved—extraction technologies require an anchor point onshore to transform marine resources into terrestrial commodities. An extensive range of facilities, such as harbors, processing plants, drying racks, and warehouses, are built manifestations of the ecological pressure on marine ecosystems ( Tavares, 2024a). They allow us to trace the connections between the built environment and fish populations. Nonetheless, a methodology for estimating such impact has yet to be established.

While cod has been studied from fish species and ecological perspective ( Kulatska *et al.*, 2025; Rose, 2007; Rose, 2008; Rose, 2019) and a variety of historical perspectives ( Campana *et al.*, 2025), their related architectural and urban dimensions remain underexplored. Architecture and urban studies document the growth of a variety of landscapes associated with fisheries, but from cultural and sociological angles. Hence, the entanglements between built environments, fish populations, and socioecological change remain insufficiently understood. Overcoming this gap requires new research methodologies capable to bridge difference disciplines and research.

This paper presents the results of a weeklong workshop held in Ísafjörður, Iceland, organized as part of the Fishing Architecture project ( Tavares, 2024b), a broader research project focused on the industrialization of fisheries along the shores of the North Atlantic exploring the interconnections between onshore fishing-related infrastructure and offshore natural resources, from 1815 to 1992. The project’s methodological backbone is structured around key analytical axes: marine ecosystems, fishing technologies, food processing, politics, and consumption habits. This framework allows us to investigate the complex interrelations between marine environments and terrestrial landscapes. For instance, aligning fluctuations of fish populations with different patterns of urban expansion and technological change can display the intricated parallel transformations happening within the two systems. Our research applies this methodology across several geographically, culturally and ecologically distinct case studies. So far, research has focused on four locations: South Brittany in France, the Sado estuary in Portugal, Ísafjörður in Iceland, and Gloucester in Massachusetts. By doing so, we aim to assess the ecological impact of fishing-related built environments and the natural resources upon which they depend. Within this context, working in Ísafjörður supported the development of experimental concepts to establish a foundation for understanding the broader patterns that emerge across other study areas.

Having developed from a simple trading post into an urban center through fishery activities during the twentieth century ( Þór, 1984), this town in the Westfjords peninsula is an ideal place to study cod in the assessment of the dynamics of fishing architecture. The paper focuses on the methodology employed to tackle these often-overlooked connections between the built environment and marine populations.

## Context

Alongside agriculture, fisheries were the predominant economic sector throughout Iceland’s history, experiencing exponential growth at the end of the nineteenth century and bolstering the numerous fishing settlements spread along its shores ( Arnason, 1995). The environmental conditions provide ideal spawning grounds during the spring season for both coastal and frontal (migratory) Atlantic cod populations, as they aggregate in pockets of marine space around the country ( Jónsson, 1996; Sólmundsson *et al.,* 2015). The largest of the spawning areas is in southwestern Iceland, where both migrant and coastal Atlantic cod spawn at varying depths ( Grabowski *et al.,* 2011; Sólmundsson *et al.,* 2015). Outside the spawning season, the northern areas provide ideal feeding grounds for migratory mature cod ( Sólmundsson *et al.,* 2015).

Ísafjörður started out as a commercial outpost that eventually took on great importance as a center of fish processing and export. The town is located on a sandbar (Eyrin) jutting out into the fjord Skutulsfjörður, which provides ideal conditions for mooring large vessels protected from the harsh conditions of the North Atlantic. From the seventeenth century onwards, this sandbar hosted a Danish trade outport which gave rise to a small community with a strong symbolic presence. In 1866, the few existing houses were granted municipal administrative status, and in 1902 the harbor welcomed its first motorized fishing boat. In the following years, Ísafjörður experienced significant urban growth, its population expanding from 830 to over 1,960 inhabitants between 1890 and 1920 ( Þór, 1984, 38). Located in the neighboring waters of the Atlantic, on the underwater cliffs of the Denmark Straight, the Halinn (Halamið) fishing grounds were identified in the first decades of the 20th century and became highly productive with the establishment of bottom trawling, until today being the most productive cod fishing ground for Icelandic trawlers. In recent decades the fishing fortunes of the Westfjords have waned, following the introduction of individual transferable quotas ( Edvardsson *et al.,* 2018). Despite still being the home base for several fishing vessels, onshore processing is now only a shadow of what it once was.

This case study is promising to understand key Fishing Architecture concepts since it provides an overview of the technological transformations from pre-industrial salted fish processing to the development of freezing technologies. These can be seen in the development of special building typologies whose change impacted the urban development. Ísafjörður proximity to cod populations preferred habitats can explain the historic development of regional fisheries. The settlement being built upon a sandspit, and its urban growth being associated with the exploitation of the marine resource (relatively impervious to other concurrent economic activities), offers a unique viewpoint to access the transformations of terrestrial forms and buildings, and access to the entangled ecological shifts happening in Atlantic waters.

The relationship between architecture and marine ecosystems, particularly in the context of fisheries, remains underexplored in both architectural and environmental humanities. Traditional architectural methodologies often isolate the analysis of the built environment from its ecological and socio-economic surroundings. Conversely, environmental assessments and marine science studies typically overlook the spatial, material, and built implications of marine ecological dynamics. Our methodological innovation lies in bridging these disciplinary gaps through a comprehensive, multi-scalar approach that integrates ecological, spatiotemporal, and cultural dimensions of fishing-related architecture and urban dynamics. Setting up this transdisciplinary framework demands interdisciplinary negotiation and methodological adjustments, finetuning language and tools to achieve sound results. By doing so, we expect to capture the reciprocity and complexity of fish as drivers of urban growth processes and coastal infrastructures as ecological agents that actively shape, and are shaped by, changing marine and terrestrial dynamics.

This is relevant considering more-than-human and socio-ecological perspectives in architectural and environmental research. Socially, this research framework supports critical reflections on the sustainability of coastal communities, the material legacy of industrial fisheries, and the adaptation of infrastructures to ecological shifts. By applying this methodology across diverse geographic case studies, we can trace shared patterns as well as site-specific responses, offering valuable insights for policymakers, architects, and communities working to reimagine the future of coastal areas. The methodological innovation we aim to contribute for must enable a deeper understanding of how fish and fisheries shape and are shaped by architecture, ecology, and society – a perspective we believe is increasingly vital in the context of environmental change and evolution of the built environment of coastal areas.

## Three approach angles

Three teams were established for the workshop, focusing on 1) urban development, 2) the cod life cycle, and 3) Icelandic fisheries. Each team was coordinated by one member of the Fishing Architecture research project with different disciplinary backgrounds: 1) architecture, 2) contextual design, and 3) marine biology. From the outline the methods to be experimented, materials were prepared in advance, which provided participants with a structured entry point and guidance. Teams were composed of a small group of master students from architecture traveling from Porto, Bergen and Reykjavik, as well as local master students from coastal and marine management and regional development, and a doctoral candidate on marine biology. The groups were formed to ensure multidisciplinarity, while also considering the participants’ personal interests and competences.

The work ran parallel with lectures on regional development in fishing coastal communities ( Kokorsch & Benediktsson, 2018); the history of the Icelandic cod fisheries and settlements ( Edvardsson, 2010); Atlantic cod behavior, population structure, migrations, and management ( Ólafsdóttir *et al.,* 2017); archival sources and the documentation of local history ( Hreiðarsdóttir *et al.,* 2005); the distribution and movement of juvenile Atlantic cod; seascapes and geography ( Benediktsson & Waage, 2015); and the urban history of neighboring communities. The lecturers, as well as the local research center community, provided critical feedback on the ongoing work and preliminary results.

While the initial outline was followed, space for creativity and exploration was intentionally left open, so that groups could adapt their approach, bring in new ideas, and pursue emerging lines of thought. With this approach, we aimed to balance between methodological preparation and openness to accommodate both rigor and innovation in the research process.

### Urban development

This group based its work on archival materials, including bibliographies, maps, and photographs. A selection of objects covering an extensive period was assembled and inventoried, and the work was organized toward: a) an archival photographic survey, and b) urban development mapping.

**Figure 1.**
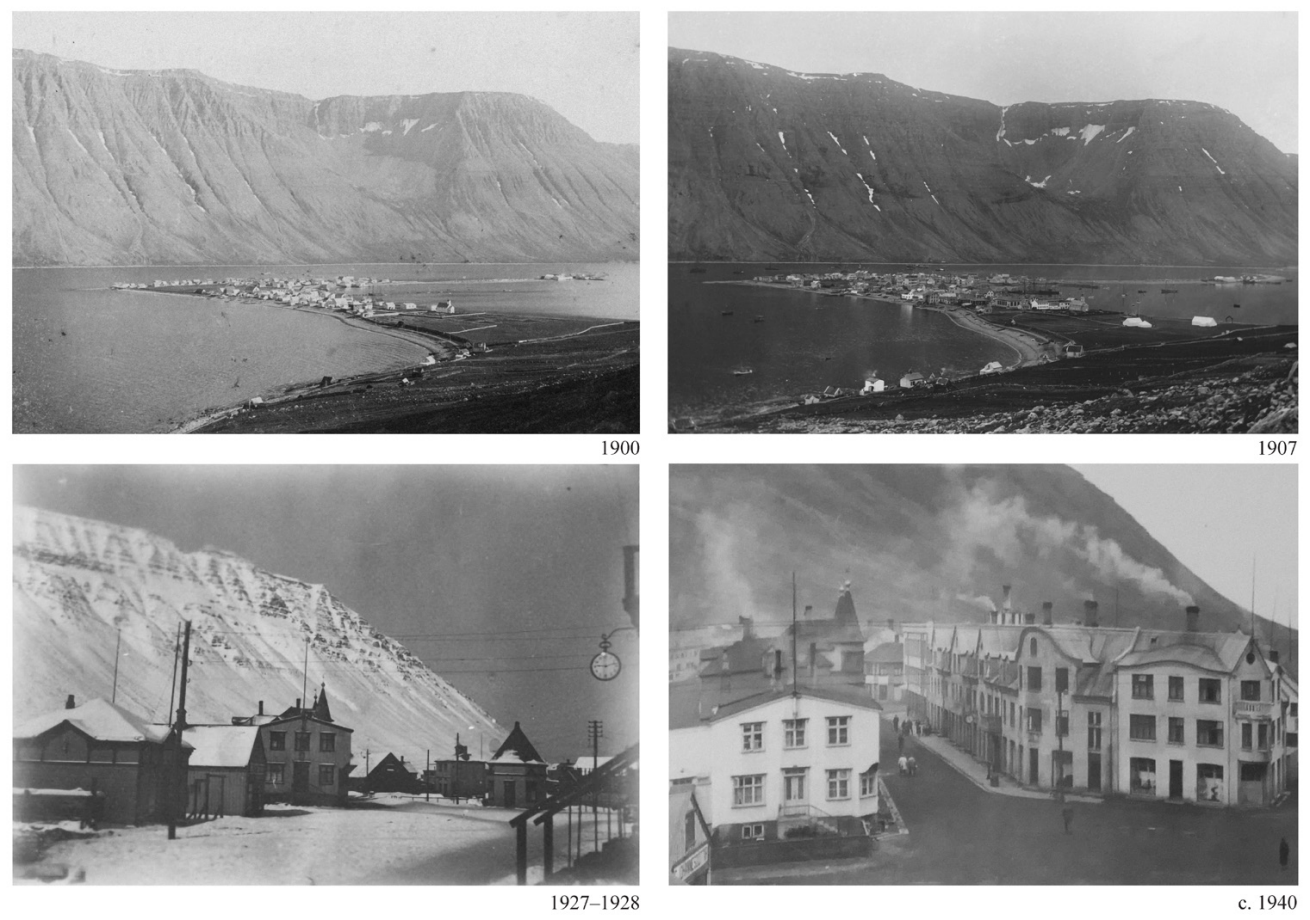
Ísafjörður, 1900 and 1907 panorama (Courtesy Safnahúsið, Ísafjörður Culture House and archives). Hafnarstræti and Silfurgata junction, 1927–1928 and circa 1940. (Courtesy Ísafjörður Regional Photo Archives).

***Archival photographic survey.*** Two sources were scrutinized to construct a geo-tagged database of historical photographs from Ísafjörður: the online database Sarpur, and a historical book on local commerce ( Halldórsson, 2007). The first task was to identify images of the main street (Aðalstræti), then expand out to the local neighborhood, and later to the entire urban area. Over 150 sources were briefly catalogued and cross-checked with a recent housing survey ( Bergsson *et al.,* 2022) and the current online property register. ^1^ Images were mapped, revealing how buildings were transformed through time, and when such transformations occurred ( Figure 1).

***Consolidated historical mapping.*** Survey plans from 1865, 1905, 1924, 1935, and 1970 were selected to depict the major urban transformations occurring within the time frame under scrutiny. After being georeferenced, it was critical to adapt their original forms to match projection systems and reference buildings and harmonize different criteria used in their original production.

**Figure 2.**
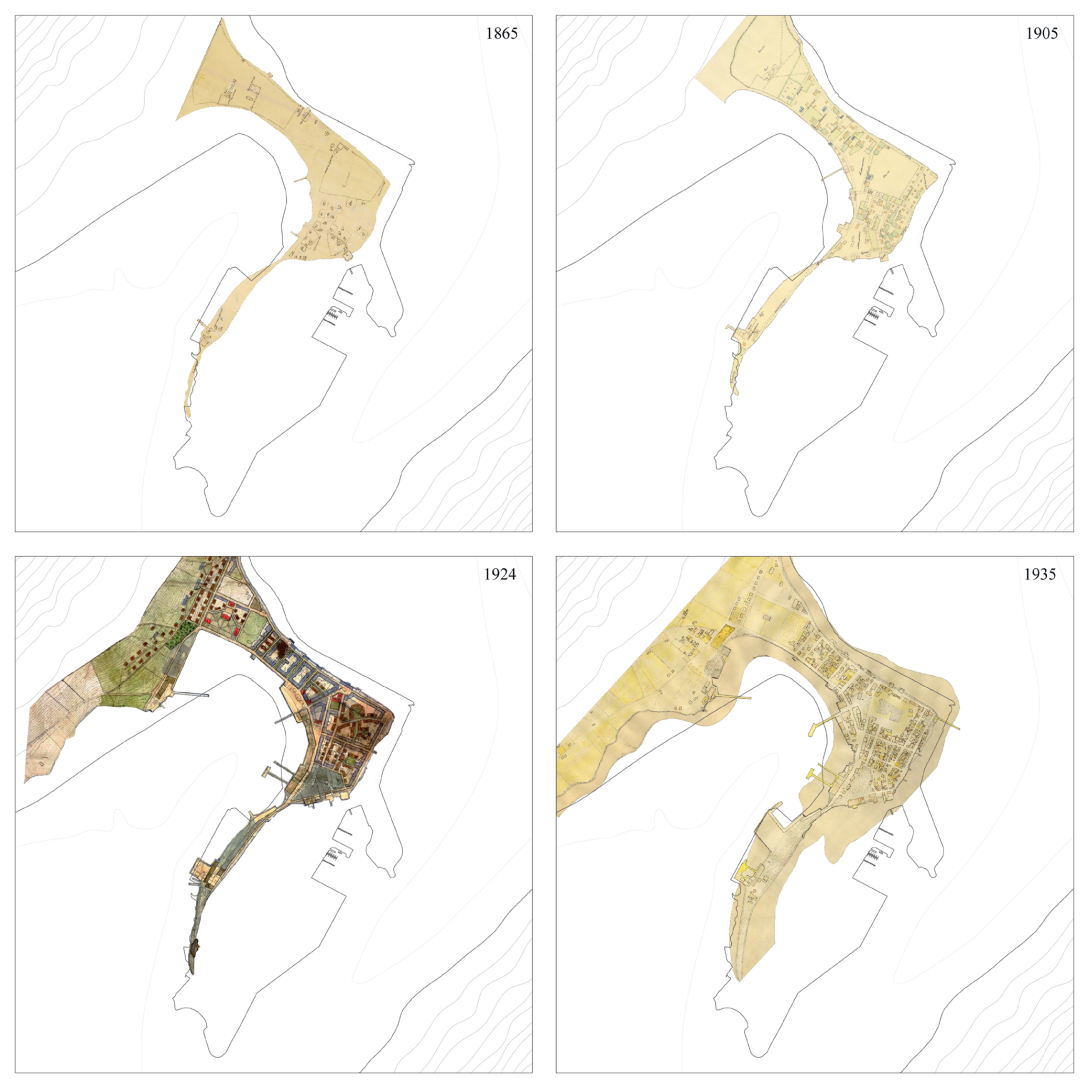
Historical maps showing the physical transformation of Ísafjörður between 1865 and 1970. Black line represents current (2024) coastline. Reference coordinates: 66.07° N, 23.12° W, WGS84. Base materials sourced from Ísafjörður Regional Archives.

The output was not a complete match: while the form and development of the Eyrin sandspit were accurate, some buildings appeared shifted ( Figure 2). The setback was overcome by adopting the 1924 urban survey, the most comprehensive of the period, as a reference to be vectorized. The maps were analyzed to obtain information on construction materials (e.g., concrete versus timber in 1924) or functions (e.g., housing, warehouses, and cod drying facilities in 1905). The outcome outlined Ísafjörður’s urban development from 1865 to 1970, highlighting the 1910s and 1920s as key periods of growth with the rise of fisheries ( Seelow, 2011, 164–67).

### Cod life cycle

The group initially selected three papers on the life cycle of Icelandic Atlantic cod ( Astthorsson *et al.,* 1994; Pálsson & Thorsteinsson, 2003; Thorsteinsson *et al.,* 2012). These papers outlined spawning grounds south of Iceland, larvae dispersal, juvenile maturation in fjords, and adults inhabiting the northern continental shelf. Complementarily, the concept of the ‘Codexian’ was introduced to bridge the disciplines of marine biology and architecture, as discussed below.

**Figure 3.**
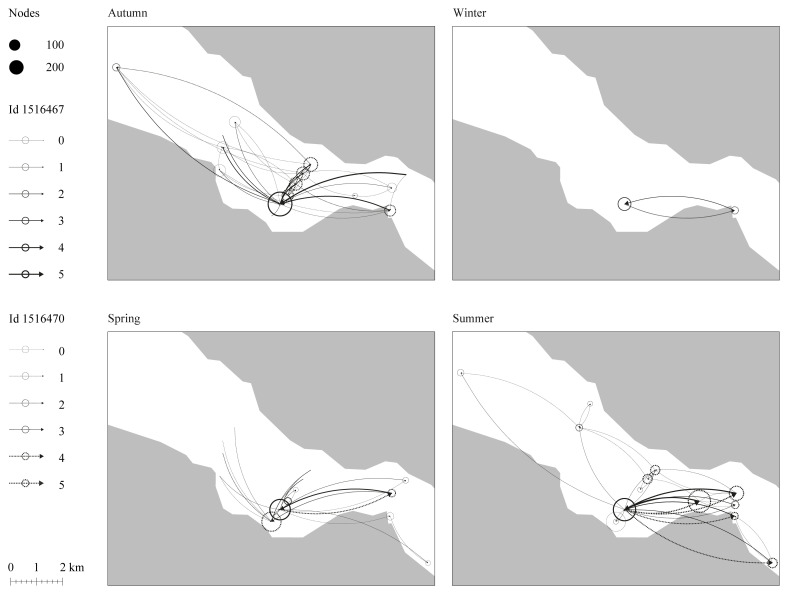
Movement network analysis of two juvenile Atlantic cod in Dýrafjörður's nursery ground over the seasons. Nodes size indicates the number of times a fish comes and goes close to a site while the arrows represent the direction and frequency of how often the individual fish moved between sites. The result shows possible sites within a nursery ground that are of interest to the fish throughout space and time.

***Movement patterns.*** As a preliminary approach to the work, three spatial and temporal scales of Atlantic cod movement and behavior were discussed: daily, seasonal, and across their development from larvae to adult. The team decided to focus on individual cod instead of populations to grasp the dynamics of cod movement and behavior for different body sizes and ecotypes. Acoustic telemetry data for the movement and behavior of two Atlantic cod juveniles (1–3 years aged) within a nearby fjord (Dýrafjörður) across different seasons were observed. ^2^ The choice of the individuals was made from a sample being tracked within the framework of one of the workshop participant doctoral research, hence the choice was dictated by data availability. The team compared individual movement over time, considering water temperature as a factor likely to influence their choices ( Figure 3). Although not conclusive, the data on cod movement patterns allowed to explore its relation with very different datasets, such as urban development patterns.

**Figure 4.**
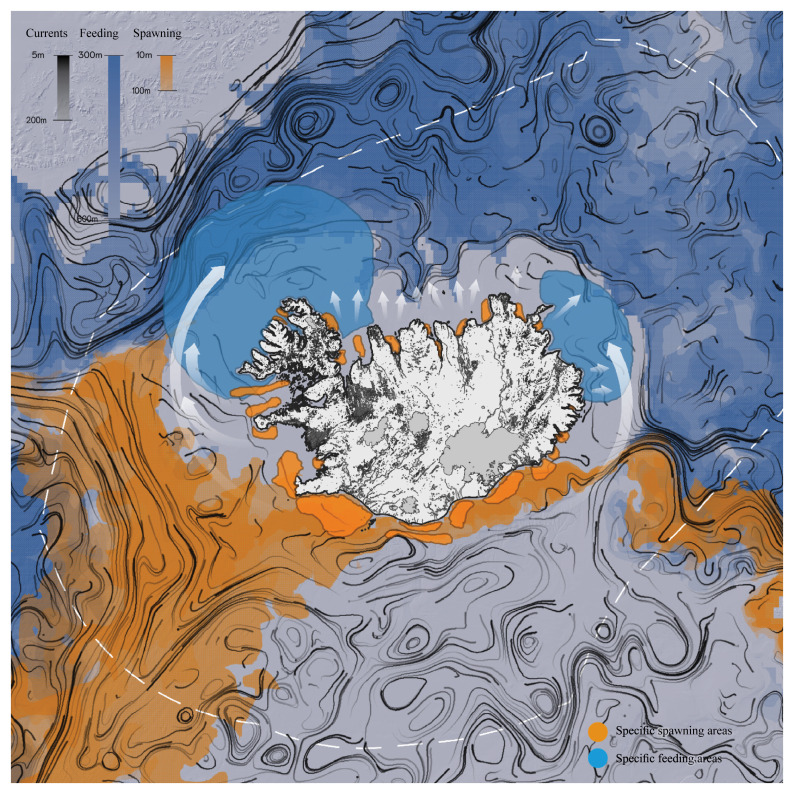
Movement patterns of Atlantic cod in the Icelandic region, spawning areas, and feeding grounds. Redrawn from Pampoulie *et al.* (2023) and overlaid with environmental data and biological factors to compute spawning and feeding grounds.

***Data assessment.*** From the literature review it was possible to identify the main parameters of habitat suitability and preferences in different stages of individual cod development. Depth, water temperature, salinity, currents, and water velocity were charted, offering visual renderings of habitat suitability for 2021. ^3^ Although inconclusive, the potential areas of interaction between the general dynamics of cod populations and their life cycle were mapped ( Figure 4).

Environmental data and map illustrations from various cod studies (e.g., Pampoulie *et al.,* 2023; Thorsteinsson *et al.,* 2012) were overlaid to understand where and when the larval life stages are likely to move around Iceland, and where adult populations are likely to move or stay resident, based on environmental (e.g., temperature, currents) and biological factors (e.g., chlorophyll, plankton) resulting in a particular spatial distribution (e.g., spawning and feeding grounds).

***Codexian.*** The Codexian—an imaginary creature—was introduced as a creative methodological and conceptual tool. This metaphorical entity symbolizes the interconnectedness between the human and marine worlds, but it can also be viewed as an attempt to create a platform of co-exploration through notions of engagement, exploration, play, and the blending of fiction and reality. The Codexian was a creature, half-cod half-researcher, equipped with elusive powers and insights that supported the construction of a fictional narrative.

Although the Codexian emerged as a creative tool, it acted as a mediator between scientific and artistic lenses. Participants enacted their rituals, using video to integrate observation, grant respect, and develop cross-disciplinary explorations. It materialized as a performance which involved actions such as casting a line with a waterproof camera and hydrophone into the sea to observe and listen to the cod. The method was an attempt—eventually closed in its own oppacity—to grasp the variables at stake in the different working time frames, underscoring the need for experimental methodologies to address the natural environment ( Hessler, 2018). More puzzling than problem-solving, the Codexian became a faithful companion to interdisciplinary research.

### Icelandic fisheries

The work of this group began with a preliminary collation of Icelandic fish landings data from 1905 to 2022, creating a dashboard to track the overall fishing activities. The objective was to support the search for historical data to fill equivalent data sets to reconstruct a historical perspective in relation to Ísafjörður’s urban development.

During the workshop, two lines of work provided consistent results: a) temporal tracing of vessel technology and capacity, and b) preliminary assessment of historical fisheries data. Relevant historical references to fishing grounds and sites were also discussed ( Edvardsson, 2010, 317).

**Figure 5.**
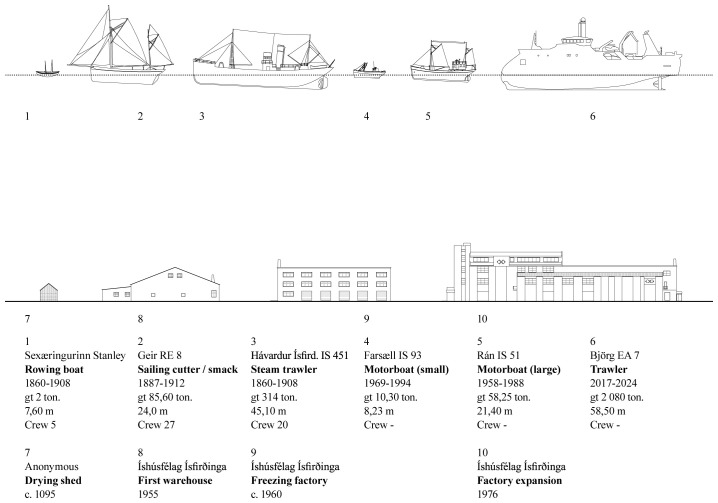
Timeline of fishing vessels and built cod processing facilities. Examples of ships registered for Ísafjörður’s cod fisheries and contemporary buildings.

***Vessel technology and capacity.*** The different vessels, their capacities, dimensions, and technological features were assessed to shed light on the major historical transformation in fishing gear and equipment used in operations based in Ísafjörður. The focus followed a historical sequence: open rowing boats; decked boats and schooners; steam and diesel motorized boats; motorized side trawlers; stern trawlers ( Jónsson, 1983; Sverrisson, 2002). The vessels emerged as the inevitable link between the terrestrial and marine ecosystems, connecting the transformations occurring in both environments ( Figure 5).

***Historical fisheries data.*** The assessment of archival data on historical fisheries allowed this group to understand what kind of data is available and to which years it applies. ^4^ Preliminary data sourced from Statistics Iceland and available in spreadsheets could be promptly processed. The most relevant pattern was the increased percentage of Icelandic Atlantic cod within the world catch, rising from a 5.95% minimum in 1968 to a peak of 22.82% in 1991. Throughout the period range, Icelandic cod saw a growth in its worldwide relevance, with a steady decrease evident elsewhere, even before the 1992 Canadian moratorium on the Grand Banks cod fisheries.

A noticeable feature was the similar patterns between herring and cod Atlantic catches, particularly in the mid-1960s and mid-1970s, a coincidence that is neutralized by the increasing economic relevance of cod as compared with herring, which was of lower value. This data could be overlapped with the archival photographic survey, considering herring’s relevance in Ísafjörður during the 1920s, in direct chronological relation to the Icelandic “herring adventure” ( Hamilton *et al.,* 2004). The statistical findings showed how herring has a lower profile within Ísafjörður’s historical narratives, which tend to emphasize cod fisheries as a major engine of its social organization.

## Discussion

Assessing the relationship between fish populations and the built environment within an ecosystem requires the consideration of a multitude of variables, including biological, ecological, environmental, and social factors. The methods adopted in the workshop helped the participants to fulfill such requirements, although its limited time frame precluded intense cross-disciplinary experimentation.

**Figure 6.**
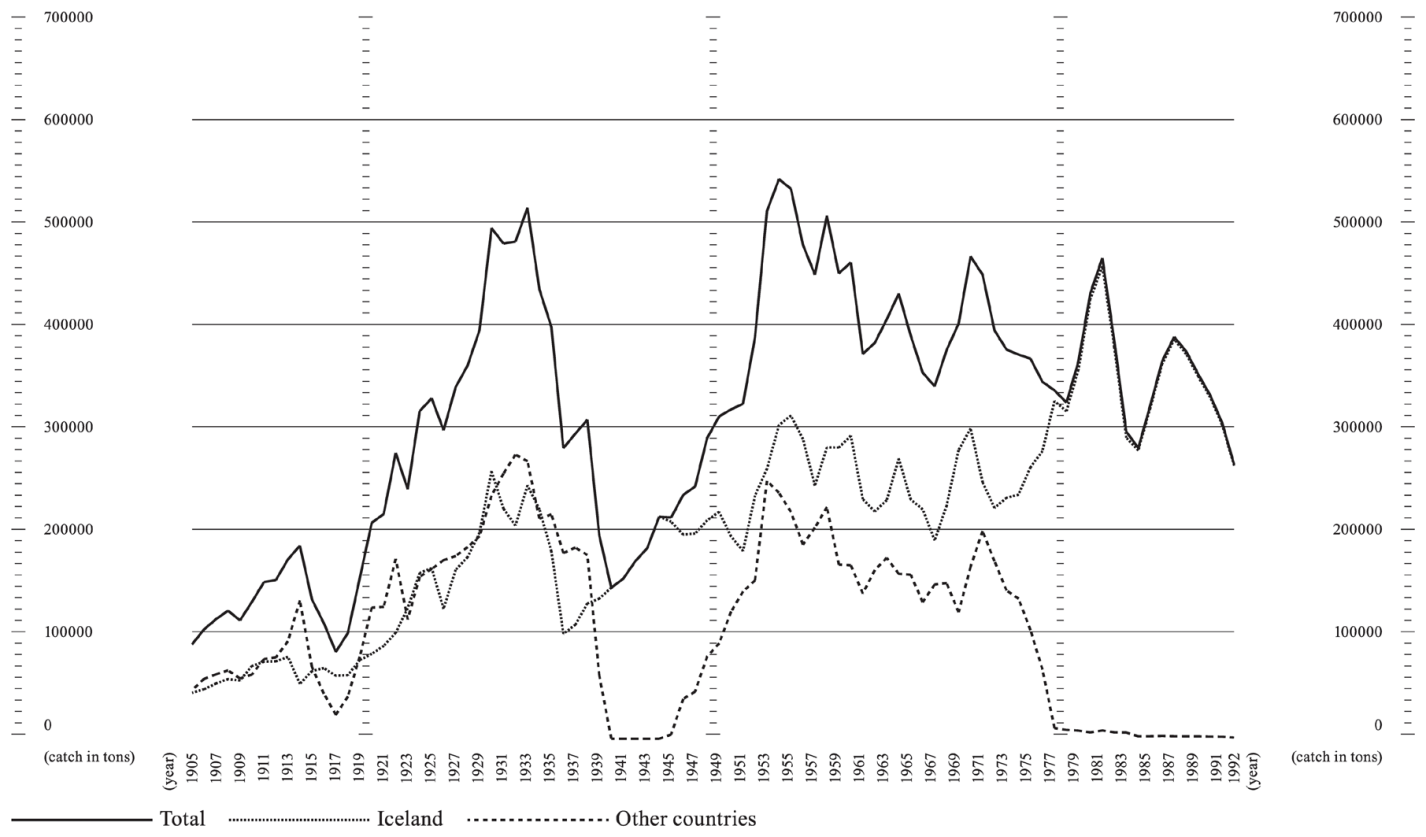
Graph showing Atlantic cod catches within ICES area Va (27.5.a, Iceland Grounds) between 1905 and 1992. Catch data is presented in tons. The lines represent catches by the Icelandic fleet (dotted line), other countries’ fleets (dashed line), and total catches in the area (solid line).

The character of the results obtained underscored the significance of visual renderings. During the discussion of the multiple research threads, the juxtaposition of the visual materials produced by each team brought evidential aspects to the fore and highlighted areas of synthesis. For example, comparing the scale of fishing gear ( Figure 5) with the scale of the urban features ( Figure 1) revealed a continuity between the marine and terrestrial forms of human activity that coincided with the amount of cod biomass extracted from its natural environment ( Figure 6).

Visual representation alone does not constitute definitive evidence of causal relationships. Instead, its exposes potential patterns, tensions, and moments of convergence that can be further interrogated through comparative analysis and future research (Bremner, 2023). Hence, it becomes a mediation tool that allows disciplinary backgrounds to work with heterogeneous data sources over a common ground. To push further research results and methods, rather than a singular method or research angle, novelty requires to bring together disparate forms of knowledge who are seldom in dialogue. And visual synthesis can propel the combination of multiple datasets and disciplinary perspectives through collaborative research. It adds that there are secure methods to combine visual outputs and quantitative data ( Baxter, 2025).

When dealing with marine systems and terrestrial landscapes, representation translates concrete dynamic processes into abstract and stable forms: the specific variables of cod behavior are represented as something fixed. Objective representations allow us to materialize connections and grasp possible understandings of the elusive relationship between fish populations and built environments. During the workshop, this possibility became obvious through an objectification of the sheer scale of cod spawning areas and feeding grounds in relation to Iceland’s land areas.

The workshop also reinforced the idea of technology as a connector between the different lines of inquiry, environmental conditions, and ecological transformations. Based on the technology deployed in fisheries but also on the methods used to acquire knowledge, such as Automatic Identification Systems, the buoys used to triangulate, record, and transmit fish-tagging devices, or access to fish-landing statistics, technology explains many of the interconnections we want to highlight and understand. Nonetheless, the fact that the prior relations persist beyond major technological shifts makes it clear that technology is no more than a link within a more complex chain of factors and events.

One inconclusive question was how to address the relationships between individual cod behaviors and population patterns. The history of urban dynamics is parallel to the economic data of fish landings, yet it is seldom sufficient to enable us to grasp a coherent picture of fish at sea. The cod life cycle is regular, but variations do exist in age and size at maturity between different groups and regions, influenced by environmental conditions. There are both abiotic (e.g. temperature) and biotic (e.g. prey availability) factors that influence fish distribution and the timing of movement events, contributing to life-history stages, spawning grounds, foraging areas, and nursery habitats ( Buchan *et al.*, 2020; Harley *et al.*, 2006; Rezek *et al.*, 2020; Robichaud & Rose, 2004). Moreover, the genetic diversity among cod ecotypes is complex where the differentiation of coastal and frontal cod in Iceland is under continued investigation, suggesting differences in movement and behaviour among ecotypes and linked with genetics (e.g., Arnason *et al.,* 2009; Beukeboom *et al.*, 2022; Beukeboom *et al.*, 2023; Pampoulie *et al.,* 2008; Pampoulie *et al.,* 2023).

The workshop results suggested a temporal mismatch in the linkage between sea and land. Ísafjörður illustrates how the spatial overlapping of urban dynamics, marine ecosystems, and cod populations does not coincide with their chronological coincidences. Its physical location is close to cod feeding grounds and to the highly productive Halinn grounds, where the continental shelf slopes steeply down into the deep ocean of the Denmark Strait. Although it was a historic trading post, Ísafjörður’s urban growth was due to motorized fisheries and the cod economy that developed in the early twentieth century. As one can see in the chronology of urban development ( Figure 2), transformations on land took time, and in decades past, urban visions were adjusted, abandoned, and redefined. Yet, the cod life cycle remained stable, from the larval to juvenile stages and on to maturity and senescence ( Figure 4). The cod’s rhythms do not match the architectural, social, and terrestrial transformation cycles. Further research following the preliminary results that came out can be tracing such chronological matches and mismatches within a specific chronological time frame. Within the framework of the Fishing Architecture research such hypothesis was further researched in the Brittany case study ( Tavares & Nouvet, 2025).

Further data acquisition and integration is required. Yet, at this stage of the research, our focus has been on establishing a robust methodological framework and clearly presenting the distinct layers of data that will feed into future synthesis. The Icelandic case study presented here is a foundational project to future integration of data and cross-case comparison with other Fishing Architecture case studies. The patterns, tensions, and convergences identified so far will serve as critical points for a broader socioecological research endeavor.

## Conclusions

While operating with fragmented data retrieved from existing literature, the workshop clearly revealed the potential that visual representations have for addressing the dynamic relationship between cod, construction, and communities. It allowed to assess the availability of data on a) fish landings and the national economy, b) the environment and physio-chemical conditions in the ocean, c) urban development, and d) the local economy. Our experimentation with different representation methods yielded approaches to e) mapping, f) statistics, and g) ecosystems. The workshop generated a local historical outline and references to further studies on the existing background. With respect to fish biology and behavior, it provided general descriptions of cod at the levels of species, population, ecotype, and individual, suggesting strategies that can be used in representing and tracking the multiple scales involved. With respect to the chronological framework of the research, which runs from 1815 to 1992, it allowed us to narrow down the time frame of the Ísafjörður case study to the period between 1897 and 1942.

The main outtake of the Cod, Construction and Communities workshop was the need to further integrate data and knowledge between disciplines and research methods. Narrowing both the timeframe and the ambition of research can be a positive strategy, although it risks blurring the perception of interconnected historical sources. Ísafjörður was consolidated as a case study and further research will be developed, focusing on the assessment of cod population dynamics, urban development patterns and building typologies.

## Ethics and consent

Ethical approval and consent were not required.

## Data Availability

The data used for Figure 3 represents a small subset of acoustic telemetry tracking data collected from two individual fish within a fjord system equipped with an acoustic receiver array. This subset includes date and timestamps, fish IDs, station IDs, and corresponding latitude and longitude coordinates. The full dataset is not currently available via online open access, as it is part of a larger ongoing tracking project. Once data collection is complete, the full dataset will be made available through the Ocean Tracking Network Data Portal. In the meantime, readers or reviewers who wish to access the subset data may contact Michelle Valliant, PhD Candidate at the University of Iceland, via email at miv1@hi.is. The data used for Figure 6 is based on the dataset Catches of Atlantic cod in Icelandic waters in the 5a area, 1905–2019, available at https://doi.org/10.5281/zenodo.15168429 ( Directorate of Fisheries, 2025). This dataset was derived from the database of the Icelandic Directorate of Fisheries ( https://statice.is/statistics/business-sectors/fisheries/), accessed on March 20, 2024. Data are available under the terms of the Creative Commons Attribution 4.0 International license (CC-BY 4.0).
