# Supplementary material for: Cod, construction, and communities: Relations between fish and architectural history in Ísafjörður, Iceland
Source: Open Res Eur. 2025 Sep 22;5:136. Originally published 2025 May 19. [Version 3] doi: 10.12688/openreseurope.20177.3 (PMC12365596; doi:10.12688/openreseurope.20177.3)
Supplement: Peer review history

# Reviewer report: Reviewer response for version 3

**DOI:** 10.21956/openreseurope.23090.r60894

**Published:** 2025-09-25

**Authors:** Preeti Kumari

**Affiliations:** 1 Amity University, Ranchi, Jharkhand, India

The author has made substantial changes in the manuscript. The manuscript may be accepted for indexing.

Is the case presented with sufficient detail to be useful for teaching or other practitioners?

Yes

Is the work clearly and accurately presented and does it cite the current literature?

Yes

If applicable, is the statistical analysis and its interpretation appropriate?

Yes

Are all the source data underlying the results available to ensure full access and reuse by other researchers?

Yes

Are the conclusions drawn adequately supported by the data and analysis?

Yes

Is the background of the case’s history and progression described in sufficient detail?

Yes

Reviewer Expertise:

Ecotoxicology; Bioaccumulation; Human Health Risk; Mercury; Arsenic; Cadmium; Environmental Toxicology; Metalloids; Heavy Metals; Biomonitoring; Environmental Pollution; Heavy Metal Toxicity; Biodiversity; Heavy Metal Pollution; Toxic Metals; Heavy Metals Analysis; Heavy Metal Accumulation; Bioassessment; Microplastics; Environment; Environmental Analysis; Environmental Analytical Chemistry; Environmental Impact Assessment; Environmental Monitoring; Fish toxicity; Bioaccumulation of Heavy Metals; Biotic indices; Environmental Risk Assessment; Toxicity; Ecological Risk Assessment; Environmental Exposure; Macroinvertebrates; Environmental Remediation; Ecology; Zoology; Remediation; Bioremediation

I confirm that I have read this submission and believe that I have an appropriate level of expertise to confirm that it is of an acceptable scientific standard.

# Reviewer report: Reviewer response for version 2

**DOI:** 10.21956/openreseurope.22620.r57143

**Published:** 2025-08-22

**Authors:** Jean C.G. Ortega

**Affiliations:** 1 Environment, Soils and Land Use, The Agriculture and Food Development Authority (Teagasc), Wexford, County Wexford, Ireland

This is an interesting and well-written study. As the authors’ framework can be applied to different contexts, it is important to provide a bit more context about which stakeholders were involved in the workshops and about the development of this activity.

 Were they all researchers?

 Were only specialists composed in each group?

 Or were they composed of stakeholders with different backgrounds?

 All stakeholders participated in the research project, or were external ones involved in it?

 If the latter, how were they involved?

 Concerning the workshops, the authors wrote that research was done in parallel with lectures. Was the research process conducted with any source of moderation?

 Movement patterns: how were the individuals chosen to have their movement tracked?

Is the case presented with sufficient detail to be useful for teaching or other practitioners?

Yes

Is the work clearly and accurately presented and does it cite the current literature?

Partly

If applicable, is the statistical analysis and its interpretation appropriate?

Not applicable

Are all the source data underlying the results available to ensure full access and reuse by other researchers?

Partly

Are the conclusions drawn adequately supported by the data and analysis?

Yes

Is the background of the case’s history and progression described in sufficient detail?

Yes

Reviewer Expertise:

Ecology, Fish ecology, Freshwater ecology, Agricultural ecosystems

I confirm that I have read this submission and believe that I have an appropriate level of expertise to confirm that it is of an acceptable scientific standard, however I have significant reservations, as outlined above.

# Response

**Published:** 2025-09-02

**Authors:** Rafael Sousa Santos

**Affiliations:** University of Porto Faculty of Architecture, Porto, Porto District, Portugal

A paragraph was added to expand on the workshop development and stakeholders, addressing the relevant questions highlighted in the review.

# Reviewer report: Reviewer response for version 2

**DOI:** 10.21956/openreseurope.22620.r57148

**Published:** 2025-08-19

**Authors:** Preeti Kumari

**Affiliations:** 1 Amity University, Ranchi, Jharkhand, India

The manuscript entitled “ **Cod, construction, and communities: Relations between fish and architectural history in Ísafjörður, Iceland” **provides the information about how urban development in Ísafjörður is intertwined with cod fisheries, using interdisciplinary and visual methods to study past and present dynamics, and suggesting approaches for future research. The research work is unique and has the potential to index in the journal. However, there are some modifications/corrections that need to be addressed:

 Abstract section is incomplete. An abstract must contain a short intro, methodology, and a brief finding. Kindly modify it.

 Introduction needs to be modified by adding research gaps.

 Kindly add latitude, longitude, scale and direction in the figure 2 of Ísafjörður.

 Kindly improve the picture quality of Figure 3 and 6. The texts are not readable.

 Discussion part is weak and it need further clarifications supported by recent references.

Is the case presented with sufficient detail to be useful for teaching or other practitioners?

Yes

Is the work clearly and accurately presented and does it cite the current literature?

Yes

If applicable, is the statistical analysis and its interpretation appropriate?

Yes

Are all the source data underlying the results available to ensure full access and reuse by other researchers?

Yes

Are the conclusions drawn adequately supported by the data and analysis?

Yes

Is the background of the case’s history and progression described in sufficient detail?

Yes

Reviewer Expertise:

Ecotoxicology; Bioaccumulation; Human Health Risk; Mercury; Arsenic; Cadmium; Environmental Toxicology; Metalloids; Heavy Metals; Biomonitoring; Environmental Pollution; Heavy Metal Toxicity; Biodiversity; Heavy Metal Pollution; Toxic Metals; Heavy Metals Analysis; Heavy Metal Accumulation; Bioassessment; Microplastics; Environment; Environmental Analysis; Environmental Analytical Chemistry; Environmental Impact Assessment; Environmental Monitoring; Fish toxicity; Bioaccumulation of Heavy Metals; Biotic indices; Environmental Risk Assessment; Toxicity; Ecological Risk Assessment; Environmental Exposure; Macroinvertebrates; Environmental Remediation; Ecology; Zoology; Remediation; Bioremediation

I confirm that I have read this submission and believe that I have an appropriate level of expertise to confirm that it is of an acceptable scientific standard, however I have significant reservations, as outlined above.

# Response

**Published:** 2025-09-02

**Authors:** Rafael Sousa Santos

**Affiliations:** University of Porto Faculty of Architecture, Porto, Porto District, Portugal

The abstract was updated to comply with the suggested structure. A paragraph was included in the introduction to further address research gaps. New files for figures were provided expecting better compression to be achieved in publication. Discussion was extended including recent references.

# Reviewer report: Reviewer response for version 2

**DOI:** 10.21956/openreseurope.22620.r57145

**Published:** 2025-08-12

**Authors:** Piatã Marques

**Affiliations:** 1 University of Toronto Scarborough, Toronto, Canada

I have now read the paper titled “Cod, construction, and communities: Relations between fish and architectural history in Ísafjörður, Iceland”. While I believe that understanding the dynamics between landscape, technology, and fish stocks is of increasing interest. While I acknowledge that interdisciplinary approaches are specially challenging to implement. I believe this paper has major flaws in the main goals, methodological approach, data obtained, data processing. Because of that I could hardly assess the strength of the discussion and conclusions. Please find specific comments below.

**Major broad comments**

 The main goal of this paper is confusing to me. The focus here is to describe the workshop and the process of knowledge building OR it is to propose a new methodology for integrating social and ecological data? I believe the paper falls very short on either case.

 The workshop activities and processes are poorly described. There is not enough information for the reader to understand what tools (e.g. group discussion, breakout groups) where used during the workshop to build knowledge (a flowchart would be key here). It is fundamental to explain how each tool builds onto another to integrate knowledge production (e.g. what is the role of the codexian as a tool? How was it built? How it integrates with the other knowledge brought by different teams?)  Also, it is impossible to know how the different players were involved in the process. What is the professional/knowledge holder make up in each focus team (i.e. how many fishers, researchers, industry / community members were involved in each team and how)? When a literature review was performed, authors must include the methodology for such review. Which keywords were used? How boolean operators were used? What were the criteria for inclusion/exclusion of papers? Without this information I cannot assess the scope or the depth of information produced in the workshop (e.g. is this an academic/expert perspective only or it involves local knowledge of non-academics?). It is also impossible to assess the transdisciplinary extent of the information produced. I suggest that, if the main goal of the paper is to report on the workshop, authors put considerably more effort into detailing the processes of knowledge production.

 I am very skeptical this paper presents any “methodological innovation”. It seems to me that the paper proposes the readers to eyeball and guess relationships based on visual representations of three datasets (urban development, cod life cycle and fisheries). Whether this can be considered a “methodological innovation” or “robust methodological framework” is very obscure to me. I am an advocate for qualitative research, but I am very skeptical that a visual approach is nearly enough to be used as a tool to link landscape and marine processes, as proposed here. This raises concerns about: 1-visual assessments being misleading and open to readers interpretation, 2- the approach incites the reader to visualize linear relationships between fishing technology, processing facilities, and fish stocks which may or may not be the case. I strongly suggest that, if the actual objective of the paper is to advance understanding on the dynamics between landscape, technology and fish stock dynamics, the authors use a quantitative approach and explore predictive models. Quantitative data on the number of cod processing facilities, area of urbanization, number of vessels, extent of fish movement and location should be straightforward to obtain and can be used to feed those models. That would be a much stronger approach than visual evaluations.   

**Major specific comments**

Introduction

 There is an overall lack of references. Multiple strong statements are made throughout the paper, without proper references. This leaves me wondering the extent to which the case for this paper is built on actual evidence supported by other studies. Some examples are:

- “An extensive range of facilities, such as harbors, processing plants, drying racks, and warehouses, are built manifestations of the ecological pressure on marine ecosystems.”
- “The relationship between architecture and marine ecosystems, particularly in the context of fisheries, remains underexplored in both architectural and environmental humanities.”
- Traditional architectural methodologies often isolate the analysis of the built environment from its ecological and socio-economic surroundings.
- “environmental assessments and marine science studies typically overlook the spatial, material, and built implications of marine ecological dynamics.”

Please provide more details as to how “This framework allows us to investigate the complex interrelations between marine environments and terrestrial landscapes”.

Methodology

 An n=2 can hardly help understand cod movement dynamics. Also, how this data was integrated with the landscape and vessel data is very unclear.

 Figure 4 is very confusing because it shows movement dynamics for the whole Iceland, while the paper focus on Ísafjörður. Please zoom in or circle the area of interest. Also, how his data is integrated with the landscape and vessel data is unclear to me.

Discussion

 Overall, I believe the discussion is vague and falls very short on referencing other studies. I also believe the paper does not show enough data (from its own research) to support the following statements:

 “The workshop also reinforced the idea of technology as a connector between the different lines of inquiry, environmental conditions, and ecological transformations”. What specific piece of information links technology, environmental conditions (what are they specifically?) and “ecological transformations” (what are these?).

 “One inconclusive question was how to address the relationships between individual cod behaviors and population patterns.” It is very hard to find conclusive relationships with sample size =2.

 “The cod life cycle is regular, but variations do exist in age and size at maturity between different groups and regions, influenced by environmental conditions.” How exactly the age/ size difference and influence of environmental conditions was assessed? I am assuming this is from the literature, but no references provided? What is the discussion regarding the data you collected and processed

Conclusion

 I am very concerned and profoundly disagree with the conclusions outlined in this paper. More specifically, I oppose the ideas that:

 “Our experimentation with different representation methods yielded approaches to e) mapping, f) statistics, and g) ecosystem modelling”. What are the specific contributions to “mapping’ (e.g. geoprocessing analysis, predictive spatial analysis, shape files produced)? What is the statistical approach when most of the data is qualitative? What is the ecosystem modelling approach when data on ecosystem processes is nonexistent?

 “With respect to fish biology and behavior, it provided general descriptions of cod at the levels of species, population, ecotype, and individual, suggesting strategies that can be used in representing and tracking the multiple scales involved.” Except for the two tagged cods, all fish biology data (behavior, population dynamics etc) were obtained from the literature. Also, It is not clear to me what sort of species level or ecotype data were presented in the paper.

Is the case presented with sufficient detail to be useful for teaching or other practitioners?

No

Is the work clearly and accurately presented and does it cite the current literature?

Partly

If applicable, is the statistical analysis and its interpretation appropriate?

Not applicable

Are all the source data underlying the results available to ensure full access and reuse by other researchers?

Yes

Are the conclusions drawn adequately supported by the data and analysis?

No

Is the background of the case’s history and progression described in sufficient detail?

No

Reviewer Expertise:

Urban Ecology, Fish Ecology, Socio-Ecological Dynamics

I confirm that I have read this submission and believe that I have an appropriate level of expertise to state that I do not consider it to be of an acceptable scientific standard, for reasons outlined above.

# Response

**Published:** 2025-09-02

**Authors:** Rafael Sousa Santos

**Affiliations:** University of Porto Faculty of Architecture, Porto, Porto District, Portugal

The purpose of the paper is to report and discuss ongoing work challenging methodology standards to develop interdisciplinary research. The reviewer’s questions point to established and long-term research practices, whereas the point of the workshop was to experiment on short-term untamed methodologies. There are certainly flaws, and more important, limitations to the outcomes—this paper does not aim to accurately describe the “the ecological impact of fishing-related built environments and the natural resources upon which they depend” but to expose how to imagine, test, discuss and expose unconventional methods to address the question. The methods used are not yet fully articulated, hence their assessment in systematic opposition to conventional and established research strategies is a relevant contribution to their continuous development. Hence, we do believe it is relevant to publish—and discuss—ongoing research. We are grateful for the thorough assessment of the paper and the workshop open ends. The flaws raised will indeed be valuable to further developments and will be taken up in future publications.

# Reviewer report: Reviewer response for version 1

**DOI:** 10.21956/openreseurope.21829.r54581

**Published:** 2025-06-13

**Authors:** Wiebren Johannes Boonstra

**Affiliations:** 1 Uppsala University, Uppsala, Sweden

The article "Cod, construction, and communities: Relations between fish and architectural history in Isafjodur, Iceland" provides a description of ongoing methodological innovation to capture the interlinkages between terrestrial development and use of ocean resources. The article provides a clear description and makes the plausible argument that the integration and juxtaposition of (sometimes unconventional) visualisations of data can help draw unexpected interrelations between terrestrial and marine phenomena.

 I find that the paper can be strengthened with a greater substantial discussion of the problem that the authors intend to address. Why is methodological innovation needed? Which scientific puzzle or social problem can it address?

 More explanation is needed on the "Codexian". What is this exactly? How was it constructed?

 More effort can be paid to integrating and synthesising the different sources of data. At it currently stands they are described and explained too much on their own account. I believe that linking their social and environmental dynamics in time can reveal can reveal useful information. The suggestion of a mismatch is somewhat superficial and indicates to me that the authors might need to consider more and more different data to understand how the terrestrial is linked (not mismatched) with the maritime.

Is the case presented with sufficient detail to be useful for teaching or other practitioners?

Partly

Is the work clearly and accurately presented and does it cite the current literature?

Yes

If applicable, is the statistical analysis and its interpretation appropriate?

I cannot comment. A qualified statistician is required.

Are all the source data underlying the results available to ensure full access and reuse by other researchers?

No source data required

Are the conclusions drawn adequately supported by the data and analysis?

Yes

Is the background of the case’s history and progression described in sufficient detail?

Partly

Reviewer Expertise:

Environmental sociology; Rural sociology; Sustainability science

I confirm that I have read this submission and believe that I have an appropriate level of expertise to confirm that it is of an acceptable scientific standard, however I have significant reservations, as outlined above.

# Response

**Published:** 2025-07-07

**Authors:** Rafael Sousa Santos

**Affiliations:** University of Porto Faculty of Architecture, Porto, Porto District, Portugal

We are very grateful for the feedback received, whose comments contributed to clarify aspects of the paper. Paragraphs in Introduction and Context sections were added to expand the information provided and respond to the main questions raised. Due to the length of the paper, the explanation of the need for methodological innovation and the scientific and social challenges addressed were kept synthetic, since a proper answer would diverge the focus of the paper from the explanation of the experimented methodology. A more detailed reference to the research project that was added offers access to the ongoing research production where the topics of relevance are fully addressed. More explanation on the Codexian was added. Minor adjustments were made to captions to Figure 4 and section 3.2.b. A paragraph was added in the discussion to address the question on data integration and synthesis. The comments on the superficial quality of the temporal mismatch were also addressed, simplifying the discussion and referencing further work. Conclusions were expanded to identify lessons learnt and next steps.

# Reviewer report: Reviewer response for version 1

**DOI:** 10.21956/openreseurope.21829.r54589

**Published:** 2025-06-09

**Authors:** Poul Holm

**Affiliations:** 1 History, The University of Dublin Trinity College, Dublin, Leinster, Ireland

The Architecture Follows Fish project is a pathbreaking and promising attempt to build a dialogue between architecture and fish biology, drawing on relevant historical data, including town and harbour plans and visual information. The case study is a concise and instructive example of how such a dialogue is being built. As such this is a valuable and publishable report.

 The paper is written from a perspective that is clearly coming from within the project. To an outside perspective, it is hard to understand exactly what is being attempted and achieved in the case study. I suggest that the authors expand the Context by introducing in a paragraph the wider project and how this case study fits the overall project design. The Conclusion may identify lessons learnt and next steps.

 It is important that the data underlying the results be published within the timeframe suggested in the paper.

Is the case presented with sufficient detail to be useful for teaching or other practitioners?

Yes

Is the work clearly and accurately presented and does it cite the current literature?

Yes

If applicable, is the statistical analysis and its interpretation appropriate?

Yes

Are all the source data underlying the results available to ensure full access and reuse by other researchers?

Partly

Are the conclusions drawn adequately supported by the data and analysis?

Yes

Is the background of the case’s history and progression described in sufficient detail?

Partly

Reviewer Expertise:

marine environmental history

I confirm that I have read this submission and believe that I have an appropriate level of expertise to confirm that it is of an acceptable scientific standard.

# Response

**Published:** 2025-07-07

**Authors:** Rafael Sousa Santos

**Affiliations:** University of Porto Faculty of Architecture, Porto, Porto District, Portugal

We are much grateful for the feedback. The manuscript was revised to improve clarity and address key points raised. The Introduction and Context sections were expanded, and a reference to the broader research project was added to contextualize the scientific and methodological challenges discussed. A new paragraph in the Discussion addresses data integration and synthesis, and the issue of temporal mismatch was clarified and linked to ongoing work. The Conclusions were updated to highlight lessons learned and outline future steps.
